# A Text-book of the Theory and Practice of Medicine by American Teachers

**Published:** 1894-03

**Authors:** 


A TEXT-BOOK OF THE THEORY AND PRACTICE OF MEDICINE BY AMERICAN TEACHERS. Edited by William Pepper, M. D., LL. D., Provost and Professor of the Theory and Practice of Medicine and of Clinical Medicine in the University of Pennsylvania. In two volumes. Illustrated. Vol. II. Philadelphia: W. B. Saunders, 925 Walnut Street. 1894. Price per volume: cloth, $5; leather, $6; half-Russia, $7.

The first volume of this fine work was reviewed in the pages of THE HOMŒOPATHIC PHYSICIAN in the number for April, 1893, page 251.

The present volume contains nearly eleven hundred pages, of which three hundred and twenty-five pages are from Dr. Pepper's own pen. It will be remembered that in the preceding volume there were two hundred pages from the pen of this extraordinary man. It has thirty-eight chapters devoted to such diseases as rickets, obesity, biliary lithiasis, gravel, diabetes, gout, rheumatism, blood diseases, diseases of the heart, diseases of the nose, larynx, bronchi, pleura, lungs, kidneys, mouth and tongue, pharynx, tonsils, œsophagus, peritoneum, intestines, liver, and pancreas.

The first chapter is upon Bacteriology, that much-discussed and yet obscure subject. It was written by Dr. William H. Welch. The volume is interspersed with plain and colored illustrations and two or three half-tone plates taken from photographs. The letter-press is beautiful and the whole work a magnificent example of bookmaking. The entire book, including the inserted colored plates, was printed on new presses recently erected especially for this purpose. The second volume has been much delayed in appearing, largely owing to the impossibility of getting the manuscript of Dr. Pepper's contribution. His busy life as a practitioner of medicine, and as general manager of the vast organization of the University of Pennsylvania, almost prevented his devoting any time at all to the portion he had planned to write. Hence the late appearance of the work.

The favorable opinion of the first volume, in a previous review, of April, 1893, above referred to, is equally applicable to this one. The two constitute a truly noble monument to Dr. Pepper and his co-laborators.

Whoever would get the latest and most comprehensive views upon pathology of the best minds in the profession cannot do better than to invest in this magnificent work.

